# The Scotch Medical Schools

**Published:** 1896-09-05

**Authors:** 


### The Scotch Medical Schools.

### MEDICAL STUDY IN SCOTLAND.

The most striking feature of medical education in Scotland is the relation between the teaching staff at the hospitals and the universities which grant the degrees. Except at Newcastle-upon-Tyne, we have nothing like it in England. There can be but little doubt that such a system, by which the students are enabled to know and be known by their examiners, however strictly the examinations may be carried out by aid of outside examiners, must, to the students, be economical of mental toil; and it is not difficult to see that in view of the impossibility of a student knowing everything, thoroughness, so far as teaching goes, is more likely to be attained when the teacher knows exactly how far he intends to carry his examination than when he is forced, as is too often the case according to the English system, to teach a bit of everything, lest peradventure the curriculum and the examination should not tally.

The next thing that strikes one is the character of the teaching, which in most of the schools is really good. Perhaps it is somewhat narrow, but really, except for the chance of being floored by the scattered questions of an omniscient examiner, as examinations are made in London, it is probably best to know a little well, and teachers can do their work better thus than where they have to battle with a sea of knowledge. In any case there can be no doubt that the mode of teaching current in Scotland, and its tendency to make the lectures and the notes thereof suffice, without being supplemented by profound book reading, does turn out students who have very crisp conceptions regarding the matters they have been taught.

There are also schools in Scotland which are not connected with the universities, but they maintain much the same character of teaching. There is a tradition that student life in Scotland is much cheaper than it is in England. It certainly used to be, and no doubt the fees are less, but times are rapidly changing. The traditional sack of meal is not popular, and it is almost as easy to spend money in Edinburgh or Glasgow as in London. Nevertheless, and perhaps we may specially instance Aberdeen, there is probably a larger leaven of hard-headed, economical young men in the Scotch schools than in the English ones, and thus a youth who chooses to be careful need not become an outsider, and hospital and college fees are less than in some of the English schools.

No doubt the schools of Scotland are to a large extent organised for Scotchmen, a hard and clever race, and it must not be too readily assumed that the same mill will with equal readiness grind Englishmen into doctors; but there is no doubt that a splendid medical education is offered by the Universities of Scotland for those who are able to assimilate their advantages.

The University of Edinburgh is a very large one, with an immense number of students attending its various departments, who are drawn to it from all countries by its well deserved fame, and every possible opportunity is offered to the medical student to obtain a thoroughly good medical education. It should be remembered that attendance at the lectures and hospital practice of the Scotch schools qualifies also for the diplomas of the Royal Colleges in England.

### Schools of Medicine in Scotland.

Aberdeen University Medical School. The cost of matriculation, class, and hospital fees for the whole curriculum, exclusive of the fees for the degrees, is usually about £90.

Edinburgh University Medical School. Separate fees for each course; for particulars see University Calendar.

Glasgow University Medical School. See University Calendar.

School of Medicine of the Royal Colleges, Edinburgh.

Anderson's College Medical School, Glasgow.

St. Mungo's College, Glasgow.

University College, Dundee. For first and second years' students.

